# Efficacy and Safety of Efpeglenatide in Patients With Type 2 Diabetes and Obesity: A Systematic Review

**DOI:** 10.7759/cureus.77089

**Published:** 2025-01-07

**Authors:** Neha Narayan, Tejaswi Vadde, Maharshikumar Sandesara, Shravani Divity, Aiturgan Mamytova, Tugolbai Tagaev

**Affiliations:** 1 General Medicine, SVS Medical College, Mahabubnagar, IND; 2 General Medicine, CU Shah Medical College and Hospital, Surendranagar, IND; 3 General Medicine, Government Medical College, Mahabubnagar, IND; 4 General Medicine, I.K. Akhunbaev Kyrgyz State Medical Academy, Bishkek, KGZ; 5 Internal Medicine and Hematology, I.K. Akhunbaev Kyrgyz State Medical Academy, Bishkek, KGZ

**Keywords:** efpeglenatide, glp-1 receptor agonists, glycemic control, obesity, type 2 diabetes mellitus, weight loss

## Abstract

Efpeglenatide, a novel long-acting glucagon-like peptide-1 receptor agonist (GLP-1 RA), shows promise for the treatment of type 2 diabetes mellitus (T2DM) and obesity. This systematic review evaluated the efficacy and safety of efpeglenatide in patients with T2DM and obesity. Literature searches in PubMed, Scopus, and Web of Science focused on randomized controlled trials (RCTs), cohort studies, case-control studies, and longitudinal observational studies from 2019 to 2024. Eight studies met the inclusion criteria. The findings of these studies consistently indicate that efpeglenatide significantly reduces hemoglobin A1C (HbA1C), fasting plasma glucose (FPG), and body weight in patients with T2DM and obesity. Once-weekly dosing offers a convenient alternative to daily GLP-1 RAs and potentially improves adherence. Efpeglenatide also provides cardiovascular and renal benefits, particularly for high-risk patients, thus providing a comprehensive treatment option. The safety profile is similar to that of other GLP-1 RAs, with mild-to-moderate gastrointestinal side effects being the most common and a low risk of hypoglycemia, especially in patients not using insulin or sulfonylureas. Most studies show a low risk of bias and enhanced reliability. However, limitations include the need for long-term safety data and variations in study design. Future research should focus on cardiovascular outcomes, long-term safety, and improvements in quality of life to fully assess the benefits of efpeglenatide. In conclusion, efpeglenatide is a promising treatment for T2DM and obesity, offering effective glycemic control, weight reduction, cardiovascular and renal benefits, a favorable safety profile, and convenient dosing.

## Introduction and background

Metabolic disorders, such as obesity and type 2 diabetes mellitus (T2DM), pose significant global health challenges. Increased obesity rates have led to the development of T2DM, creating a need for treatments that address both conditions [1]. Traditional strategies have focused on lifestyle changes and medications to improve glucose control and reduce weight [2,3]. Addressing these disorders is critical. Obesity worsens T2DM, and managing T2DM can facilitate weight loss and improve metabolic health [4].

Glucagon-like peptide-1 receptor agonists (GLP-1 RAs) are promising candidates because of their mechanisms of action, including increased insulin production, reduced glucagon release, and slow gastric emptying, which enhance glycemic control and aid in weight loss [3]. Efpeglenatid, a GLP-1 RA, is a promising therapeutic agent for treating both obesity and T2DM. Unlike many GLP-1 RAs that require frequent administration, the once-weekly dosing of efpeglenatide may improve patient adherence. Efpeglenatide effectively reduces body weight and enhances glycemic control in patients with T2DM, highlighting its dual benefits [1,5]. It also has a favorable safety profile, with gastrointestinal side effects being the most common, which is consistent with other GLP-1 RAs [6,7].

The potential of efpeglenatide to revolutionize obesity and T2DM treatment is underscored by its dosing regimen and underlying mechanisms. Efpeglenatide significantly controls glycemia and induces weight loss, which is crucial for managing both T2DM and obesity [8]. Once-weekly administration offers a convenient regimen that may enhance patient adherence and satisfaction [9]. Additionally, studies suggest potential cardiovascular benefits, aligning with the comprehensive diabetes care goals [1].

This systematic review aimed to evaluate the efficacy and safety of efpeglenatide, a long-acting GLP-1 RA, in managing T2DM and obesity. This review examined the effects of efpeglenatide on glycemic control, weight reduction, and cardiovascular outcomes as well as its safety profile compared with those of placebo and other GLP-1 RAs to establish its role in comprehensive metabolic and diabetes management.

## Review

Material and methods

This systematic review adhered to the Preferred Reporting Items for Systematic Reviews and Meta-Analyses (PRISMA) guidelines to ensure rigor, transparency, and replicability [10].

Electronic databases, including PubMed, Scopus, and Web of Science, were systematically searched using keywords and Medical Subject Headings terms such as “efpeglenatide,” “type 2 diabetes,” “obesity,” “GLP-1 receptor agonists,” “glycemic control,” “HbA1c,” “body weight,” and “safety profile.”

The search focused on peer-reviewed publications published between 2019 and 2024 to ensure the inclusion of updated information. Inclusion criteria comprised randomized controlled trials (RCTs), cohort studies, case-control studies, and longitudinal observational studies on the efficacy and safety of efpeglenatide in adults with T2DM. Studies having both patients with or without obesity were included. Studies needed to report quantitative data on outcomes such as glycemic control (hemoglobin A1C (HbA1C) levels and fasting plasma glucose (FPG)), body weight, body mass index (BMI), lipid profile, and adverse effects. Only English-language studies involving humans were included. Studies on pediatric populations, preclinical studies, or those with insufficient outcome data were excluded.

One reviewer conducted the initial search, and duplicates were removed using reference management software (EndNote). Two independent reviewers screened the titles and abstracts for relevance, and full-text screening was performed for studies that met the inclusion criteria. Disagreements were resolved by consensus or by involving a third reviewer. The reference lists of the included articles and relevant reviews were manually searched for additional relevant studies.

Data extraction was performed independently by two reviewers using a standardized form. The extracted data included study characteristics (author, year, country, design, and sample size), participant demographics (age, sex, baseline BMI, and T2DM duration), intervention details (efpeglenatide dosage, frequency, treatment duration, and comparator information), primary and secondary outcomes (HbA1c level, FPG level, body weight reduction, BMI change, lipid profile, and cardiovascular outcomes), and safety and tolerability (adverse events). Efforts were made to contact the authors regarding missing and unclear information.

Standardized tools, including the Cochrane Risk of Bias (RoB 2.0) for RCTs and ROBINS-I for observational studies, were used to assess the quality and risk of bias in the included studies. The RoB 2.0 tool evaluated domains such as selection, performance, detection, attrition, reporting, and other biases. The ROBINS-I tool assessed similar domains, including confounding factors, participant selection, intervention classification, deviations from intended interventions, missing data, and selective outcome reporting. Two reviewers independently assessed the risk of bias, with disagreements resolved through discussion or by involving a third reviewer. Each domain was classified as low, moderate, or high risk, and the overall risk was determined for each study.

Results

**Figure 1:**
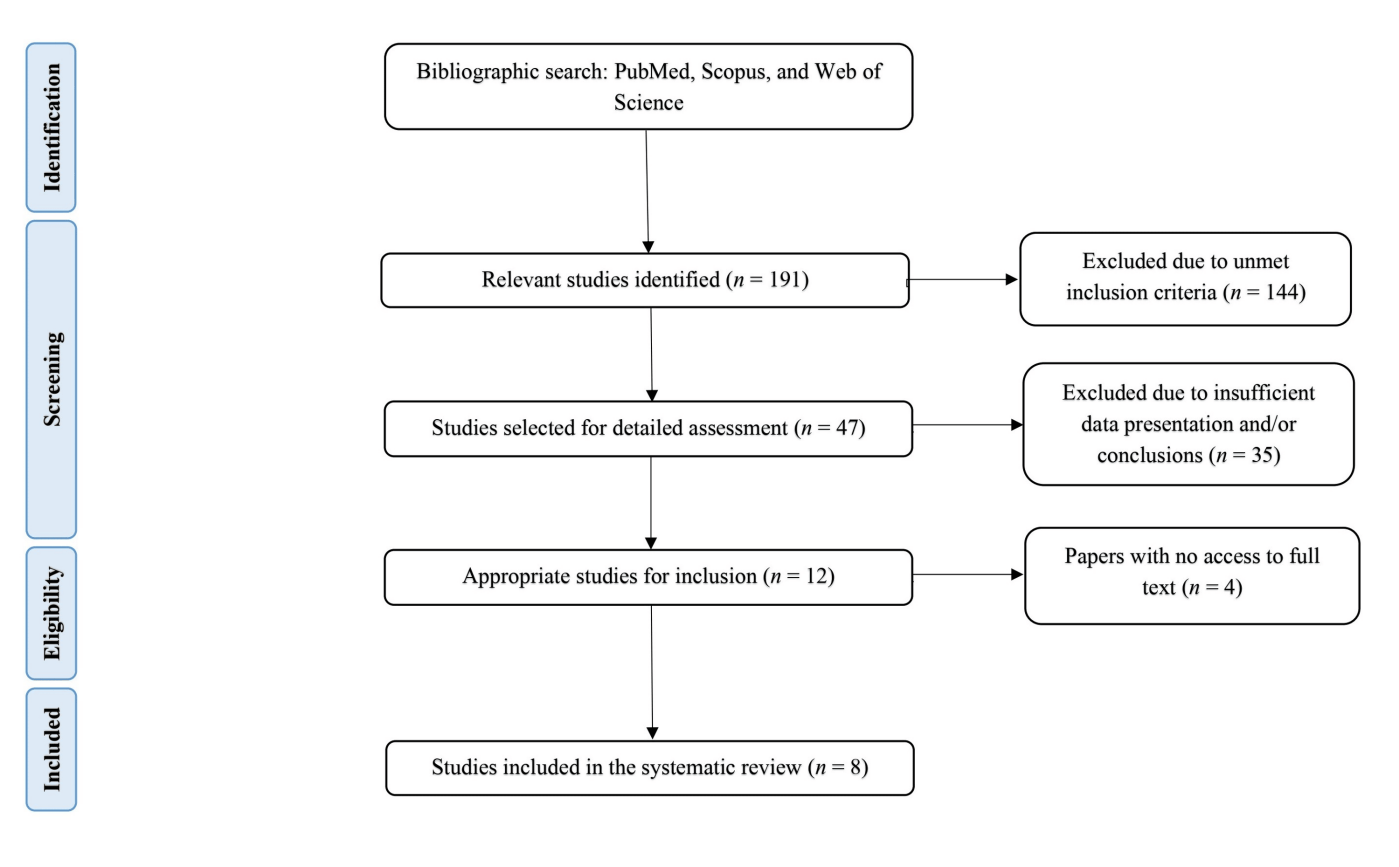
Flow diagram of literature search and study of selection for systematic review (PRISMA flow chart) PRISMA, Preferred Reporting Items for Systematic Reviews and Meta-Analyses

The initial literature search yielded 191 results, which were filtered according to specific inclusion and exclusion criteria. Of the 191 articles, 144 were excluded because they did not meet the inclusion criteria owing to inappropriate research designs (e.g., preclinical or pediatric studies), insufficient reporting of results, or lack of quantitative data on glycemic control, weight management, or safety. Some studies were inaccessible, despite institutional access requests and direct author communication. The remaining 47 articles were subjected to comprehensive analysis, of which 35 were excluded if they lacked relevant quantitative outcome data, detailed methodology or findings, or statistically supported conclusions. Of the 12 remaining articles, four were inaccessible in full text despite multiple retrieval attempts, leaving eight studies for systematic review [4,9,11-16]. Figure 1 illustrates the selection process, and Table 1 lists the eight studies selected for this systematic review.

**Table 1:** Characteristics of selected studies on the efficacy and safety of efpeglenatide in patients with T2DM and obesity T2DM: type 2 diabetes mellitus; HbA1c: hemoglobin A1c; GLP-1 RA: glucagon-like peptide-1 receptor agonist; QW: weekly; QM: monthly; QD: daily; SGLT2: sodium-glucose co-transporter-2; MACEs: major adverse cardiovascular events; RCTs, randomized controlled trials; FPG, fasting plasma glucose; BMI, body mass index

Study details	Study objectives	Study design	Intervention	Main findings
Aroda et al. [11]	This study assessed the effectiveness and safety of once-weekly efpeglenatide in patients with suboptimally controlled T2DM.	RCTs	Once-weekly efpeglenatide	Efpeglenatide demonstrated non-inferiority to dulaglutide 1.5 mg in HbA1c reduction at week 56 in the AMPLITUDE-D trial with comparable weight loss across all groups. In AMPLITUDE-L and AMPLITUDE-S, efpeglenatide significantly reduced HbA1c and weight compared with placebo. The incidence of level 2 hypoglycemia was low across all trials.
Pratley et al. [9]	This study evaluated the efficacy and safety of efpeglenatide across various participant subgroups in the BALANCE 205 study categorized according to pre-diabetes condition, BMI, and early age.	A randomized, placebo-controlled, double-blind, parallel-group trial	Efpeglenatide: 4 mg weekly for 20 weeks, 6 mg weekly for 20 weeks, 6 mg biweekly for 20 weeks, and 8 mg biweekly for 20 weeks	Efpeglenatide significantly improved the percentage of prediabetic patients attaining normal blood glucose levels and lowered HbA1c, FPG, and body weight compared with placebo. Superior weight loss was observed across all BMI and age subgroups. Most side effects were gastrointestinal, aligning with the known safety profile of GLP-1 RAs.
Frias et al. [4]	This study demonstrated that efpeglenatide significantly reduced HbA1c levels compared to placebo after 30 weeks, with additional glycemic markers and weight changes assessed at 30 and 56 weeks.	A phase 3, double-blind, placebo-controlled, multicenter, randomized trial	Efpeglenatide 2, 4, or 6 mg upto 56 weeks for QW	Efpeglenatide significantly lowered HbA1c levels at 30 weeks compared with placebo, indicating better blood sugar control. More participants on efpeglenatide reached the target HbA1c levels (<7%) than those on placebo. The doses of 4 and 6 mg of efpeglenatide also significantly reduced body weight and FPG levels.
Hompesch et al. [13]	This study assessed the impact of QW and QM administration of efpeglenatide on gastric emptying compared with daily administration of liraglutide. Evaluated the impact on glucose metabolism and islet beta-cell functionality, as well as assessed safety and acceptability.	A phase Ib, parallel-group, exploratory, single-center, randomized, study	Efpeglenatide 6 mg QW for one month. Efpeglenatide 16 mg QM for three months. Liraglutide 1.8 mg QD for one month	Efpeglenatide administered at 6 mg/QW demonstrated similar efficacy to liraglutide in prolonging gastric emptying time at peak concentrations, whereas the 16 mg/QM dose was less effective. Nonetheless, both efpeglenatide dosing regimens showed comparable or superior glucometabolic effects and enhanced beta-cell function relative to liraglutide.
Lam et al. [14]	This study examined the effect of efpeglenatide in patients with T2DM and cardiovascular or kidney disorders, focusing on MACEs, extended MACEs, composite renal endpoints, and MACEs or death. This study also assessed the combined effects of SGLT2 inhibitors and efpeglenatide on clinical outcomes, and whether the efficacy and safety of efpeglenatide were influenced by concurrent SGLT2 inhibitor use.	A randomized, stratified, placebo-controlled trial	Efpeglenatide was administered QW. Some participants also received SGLT2 inhibitors, although the specifics of the frequency, duration, and dosage were not clearly defined	Efpeglenatide reduced major adverse cardiovascular events, enhanced renal outcomes, and decreased MACEs and mortality among patients with T2DM. Its efficacy and safety remain consistent with or without SGLT2 inhibitors. Efpeglenatide uniformly reduced blood pressure, body weight, low-density lipoprotein cholesterol, and urine albumin-to-creatinine ratio independent of SGLT2 inhibitor use.
Del Prato et al. [15]	This study identified the optimal QM dosage of efpeglenatide in patients with T2DM who were inadequately controlled with metformin.	A phase 2, multicenter, randomized, double-blind, parallel-group, placebo-controlled clinical trial	Efpeglenatide administration began with 4 mg of QW for a four-week titration phase, followed by an initial QM dose of 8 mg. Thereafter, the patients received 8, 12, or 16 mg QM. Metformin dosage remained unchanged throughout the study period.	QM efpeglenatide significantly improved glycemic control and reduced body weight in patients with T2DM inadequately treated with metformin. Adverse effects, primarily gastrointestinal, were consistent with the known GLP-1RAs safety profile. QW administration may provide more stable pharmacokinetics and better tolerability than QM administration.
Rosenstock et al. [12]	This study assessed the efficacy, safety, and tolerability of QW efpeglenatide in patients with T2DM.	A randomized, placebo-controlled, double-blind, parallel-group, dose-ranging study	Efpeglenatide QW in doses of 0.3, 1, 2, 3, or 4 mg for 12 weeks; Liraglutide QD up to 1.8 mg for 12 weeks.	QW efpeglenatide significantly lowered HbA1c levels in patients with early-stage T2DM compared to placebo. Doses of 3 and 4 mg resulted in notable weight loss compared with the placebo group. The safety profile matched that of other GLP-1 RAs, with no neutralizing antibodies detected.
Pratley et al. [16]	This study assessed the safety, tolerability, and efficacy of weekly or biweekly subcutaneous efpeglenatide for reducing body weight in overweight or obese patients with comorbidities, excluding diabetes, over a 20-week period.	A phase 2, multicenter, randomized, double-blind, placebo-controlled, parallel-group trial	Efpeglenatide 4 and 6 mg QW and 6 and 8 mg QM were administered with a hypocaloric diet (~500 kcal/day deficit) for 20 weeks	Over 20 weeks, efpeglenatide significantly reduced body weight, waist circumference, and BMI in overweight or obese individuals without diabetes. It also improved metabolic indicators such as glucose metabolism and lipid profiles, showing good tolerability.

Studies have indicated that efpeglenatide, a novel, long-acting GLP-1 RA, significantly benefits patients with T2DM and obesity. RCTs have shown its superiority over placebo in lowering HbA1c levels, reducing body weight, and decreasing FPG levels [4,11,12]. These effects have been consistent across diverse patient populations, including individuals with prediabetes, various BMI ranges, and different ages [9]. Efpeglenatide enhances beta-cell function and achieves similar or better glucometabolic outcomes than those of liraglutide [13]. Its effectiveness and safety profile remain consistent even with the concurrent use of sodium-glucose co-transporter-2 (SGLT2) inhibitors [14]. Gastrointestinal side effects are the most common adverse events but are generally mild to moderate and temporary [15,16]. Therefore, efpeglenatide may be a valuable therapeutic option for T2DM and obesity.

Research indicates that efpeglenatide effectively manages blood sugar and body weight, making it a promising treatment for T2DM and related metabolic disorders. Its benefits extend beyond traditional glucose targets and include obesity, heart disease risk, and kidney function.

Aroda et al. found that efpeglenatide significantly reduced HbA1c levels and body mass, highlighting its effectiveness in glycemic control and weight management [11]. The AMPLITUDE-D trial showed performance comparable to that of dulaglutide, with an average weight loss of approximately 3 kg over 26 weeks. Its once-weekly dosing schedule increases its practicality for patients requiring simplified treatment plans. The AMPLITUDE-L and AMPLITUDE-S studies also reported significant reductions in HbA1c, FPG, and body weight compared with those of the placebo, with low rates of hypoglycemia, particularly in patients not using insulin or sulfonylureas [9].

Pratley et al. supported these findings, demonstrating improved glycemic control and weight loss across various patient groups, including those with prediabetes, varying BMIs, and different ages [9]. This suggests that efpeglenatide has broad applicability in the treatment of diabetes. Frias et al. observed dose-related benefits, with higher doses (4 and 6 mg) achieving greater reductions in HbA1c and body weight compared with those in placebo [4]. These findings highlight the potential for tailored treatments that meet individual patient needs, ensuring both effectiveness and safety.

Efpeglenatide has significant effects on gastric emptying, beta-cell function, and cardiovascular markers beyond its glycemic control capabilities. Hompesch et al. reported that efpeglenatide slowed gastric motility similarly to liraglutide, aiding in weight loss. The study also showed improvements in glucose metabolism and beta-cell function, thereby supporting pancreatic health [13].

The AMPLITUDE-O trial analysis of Lam et al. highlighted the cardiovascular benefits of efpeglenatide, noting a 27% reduction in major adverse cardiovascular events among patients with high-risk T2DM, especially when combined with SGLT2 inhibitors [14]. This suggests a synergistic effect between GLP-1 RAs and SGLT2 inhibitors enhance blood sugar control, reduce cardiovascular and renal risks, and aid weight management through distinct mechanisms. This multifaceted approach aligns with the complexity of T2DM, particularly in patients with cardiovascular and renal risk factors.

The therapeutic potential of efpeglenatide is further evidenced by its efficacy relative to that of other GLP-1 RAs. Del Prato et al. reported significant HbA1c and weight reductions in obese patients with T2DM treated with efpeglenatide, comparable to the outcomes observed with liraglutide and semaglutide [12]. The EXCEED 203 trial by Rosenstock et al. confirmed these findings, showing that efpeglenatide achieved glycemic control similar to liraglutide, with a safety profile characterized by mild-to-moderate transient gastrointestinal side effects [15].

Pratley et al. explored the effects of efpeglenatide in non-diabetic obese adults in a phase II trial, demonstrating significant weight loss and suggesting its potential for treating obesity as an independent metabolic disorder [16]. These results expand the application of efpeglenatide and present it as a viable option for weight management in non-diabetic individuals.

The tolerability and safety profile of efpeglenatide resemble those of other GLP-1 RAs, with common gastrointestinal side effects such as nausea and diarrhea. Trujillo et al. [3] observed that these effects are typically mild-to-moderate and decrease over time. The risk of hypoglycemia is low, particularly in patients not using insulin or sulfonylureas. These safety features make efpeglenatide a well-tolerated option for the long-term management of metabolic disorders.

Once-weekly efpeglenatide enhances patient compliance and convenience, particularly for patients with complex regimens or adherence issues. Its dual role in regulating blood sugar and controlling weight supports holistic diabetes management by addressing glycemic levels and reducing cardiovascular and kidney risks associated with obesity and T2DM. Further research is required to evaluate its effects on heart-related mortality, kidney function, and overall quality of life. Real-world data on adherence and effectiveness across different populations, as well as comparisons with therapies such as GLP-1 RAs and SGLT2 inhibitors, are crucial. These insights will optimize the role of efpeglenatide in diabetes and obesity management.

**Figure 2:**
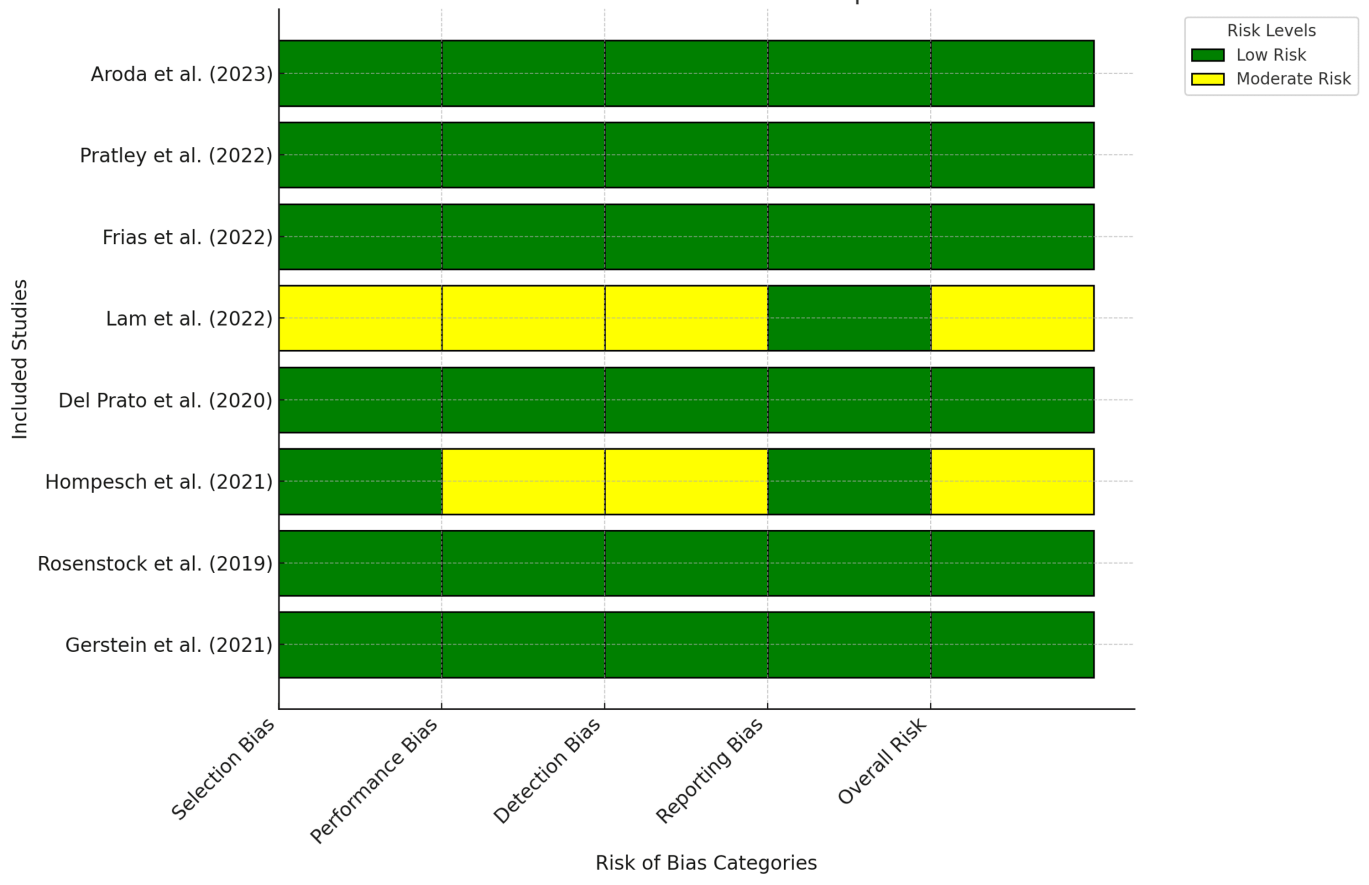
Individual risk of bias in systematic review studies on the efficacy and safety of efpeglenatide in patients with T2DM and obesity T2DM, type 2 diabetes mellitus

Most studies in the analysis exhibited a low risk of bias, enhancing the credibility of the results (Figure 2). Six of the eight studies (Frias et al., Gerstein et al., Aroda et al., Rosenstock et al., Del Prato et al., and Pratley et al.) showed low risk across all bias domains, including selection, performance, detection, and reporting, employing stringent methodologies to reduce bias and improve outcome reliability [4,9,11,12,15,16]. However, Hompesch et al. and Lam et al. showed a moderate risk in certain domains [13,14]. Lam et al. displayed a moderate risk of selection and performance bias due to inadequate randomization or blinding [13], whereas Hompesch et al. showed a moderate risk of performance and detection bias, potentially affecting outcome evaluations [14]. Despite these issues, both studies maintained a low risk of reporting bias, indicating that their overall findings remain valid. These observations suggest that most studies were methodologically sound, but future research should improve randomization, blinding, and detection processes to minimize bias and enhance reliability.

Research has demonstrated the efficacy of efpeglenatide as a GLP-1 RA for improving glycemic control, inducing weight loss, and offering cardiovascular protection. Its safety, convenient dosing, and effectiveness across diverse groups make it a valuable treatment option for T2DM and obesity. Continued research is vital to fully harness its potential and ensure optimal use in clinical practice.

Discussion

This systematic review highlights the efficacy and safety of efpeglenatide as a treatment for T2DM and obesity. The findings support the established role of GLP-1 RAs in metabolic disease management, with efpeglenatide exhibiting notable effects on glycemic control, weight reduction, and cardiovascular protection.

Efpeglenatide demonstrates significant reductions in HbA1c and FPG, consistent with the findings of Gerstein et al., who reported a 1.3% decrease in HbA1c with efpeglenatide over 52 weeks, outperforming placebo [1]. Similarly, Frias et al. observed a 1.5% HbA1c reduction over 30 weeks in the AMPLITUDE-M trial, which underscored the glycemic control potential of efpeglenatide [4]. These reductions aligned with those observed with other GLP-1 RAs, such as semaglutide, which showed similar HbA1c and FPG improvements [17].

A significant weight reduction has also been observed with efpeglenatide, with studies reporting a mean weight loss of 4.5 kg over 26 weeks [9]. This effect is consistent with findings from other GLP-1 Ras, such as semaglutide [17], highlighting the impact of efpeglenatide on weight management. This dual benefit of glycemic and weight control is critical for patients with T2DM, particularly those with comorbid obesity.

The once-weekly dosing of efpeglenatide offers a practical advantage over daily treatments such as liraglutide. The EXCEED 203 trial has shown glycemic efficacy non-inferior to that of liraglutide, with comparable safety outcomes [12]. Trujillo et al. have further highlighted the favorable safety profile of efpeglenatide, showing a similar or lower incidence of gastrointestinal side effects compared with those observed with other GLP-1 RAs [3]. With its extended half-life, which supports adherence, efpeglenatide provides a suitable alternative for patients who may benefit from a more manageable dosing schedule.

Efpeglenatide demonstrates significant cardiovascular and renal benefits in T2DM patients. The AMPLITUDE-O study by Lam et al. reported a 27% reduction in major adverse cardiovascular events, including heart attack, stroke, and cardiovascular death [11], an effect that aligns with similar cardiovascular benefits observed with liraglutide [18,19]. Additionally, efpeglenatide reduced albuminuria and improved kidney function, particularly when combined with SGLT2 inhibitors, suggesting a synergistic effect [2,20]. Gerstein et al. corroborated these cardiovascular benefits, noting fewer cardiovascular events and heart failure hospitalizations [1]. These findings align with the broader effects of GLP-1 RAs, as highlighted by Tommerdahl et al. who underscored their role in comprehensive diabetes care [2].

Efpeglenatide significantly reduces HbA1c levels, a crucial marker of long-term glucose control in patients with T2DM, as demonstrated in studies such as AMPLITUDE-O, in which several participants achieved target glycemic levels [1,14,21]. It also promotes notable weight loss, which is essential for managing obesity, a common T2DM comorbidity. Efpeglenatide-treated patients experience significant weight reduction, enhancing their overall metabolic health [22-24]. The safety profile of efpeglenatide has been extensively investigated, with common side effects, including mild-to-moderate gastrointestinal issues that typically subside over time [11,13,25]. Serious adverse events are rare, supporting its short-term and long-term safety [1,22,26]. The efficacy of efpeglenatide is comparable to that of other GLP-1 RAs but may offer advantages in dosing frequency, potentially improving patient compliance and convenience [11,21].

Although 144 studies were excluded because they did not meet the inclusion criteria and some were inaccessible due to restrictions, the included studies provided substantial evidence to meet the primary objectives, despite this limitation. Efpeglenatide's weekly dosing benefits individuals struggling with medication adherence, including those with complex treatment plans or limited access to healthcare. Aroda et al. and Frias et al. underscored the convenience of once-weekly dosing, potentially enhancing adherence and satisfaction [4,11]. Moreover, the efficacy of efpeglenatide across various BMI ranges and in patients with cardiovascular or kidney disease demonstrates its broad applicability. These features make efpeglenatide a valuable treatment for diverse patient groups, including those facing healthcare access and adherence challenges, thus supporting its clinical use.

The examined studies showed results that were likely influenced by patient factors, such as BMI and existing comorbidities. Pratley et al. found that patients with a higher BMI experienced more significant weight reduction, whereas glycemic control improvements were consistent across BMI categories with efpeglenatide [16]. Additionally, a study by Lam et al. indicated that concurrent conditions, such as cardiovascular or kidney disease, may have affected the observed benefits to cardiovascular and kidney function [14]. These findings highlight the need for personalized treatments that consider individual characteristics, such as BMI and coexisting health issues, to enhance therapeutic efficacy.

The Cochrane Risk of Bias Tool for RCTs and the ROBINS-I Tool for observational studies were used to assess bias risk. Most studies demonstrated minimal risk of bias, particularly in selection, performance, and reporting bias. Aroda et al., Pratley et al., and Frias et al. conducted robust studies using well-randomized methods, adequate blinding, and low attrition rates, yielding consistent results and bolstering confidence in their findings [4,9,11]. However, Lam et al. and Hompesch et al. showed moderate performance and detection bias risks owing to insufficient blinding and subjective outcome assessments [13,14]. In some studies, the open-label design potentially introduced a performance bias, influencing patient-reported outcomes. Some studies have also exhibited selective reporting bias, with insufficient data on secondary endpoints. Nonetheless, the overall methodological quality of the studies supports the reliability of the results.

Methodological limitations in trials with moderate bias risk, such as inadequate blinding or reporting, may have introduced variability in results, potentially exaggerating subjective outcomes such as weight loss or underreporting adverse effects [13]. However, the high-quality studies (Aroda et al. and Pratley et al.) consistently support the efficacy and safety of efpeglenatide in improving glycemic control and reducing body weight, using sensitivity analyses or subgroup evaluations to minimize bias across diverse patient populations [9,11].

The favorable side-effect profile of efpeglenatide improves adherence, particularly in patients with complex therapeutic needs. Efpeglenatide shows superior outcomes compared with existing treatments in patients with T2DM and obesity or elevated cardiovascular risk, aligning with the guidelines emphasizing comprehensive risk reduction.

Although efpeglenatide is a promising option for patients with T2DM and obesity, the current research has limitations, particularly regarding long-term safety data and study design variations. Future studies should focus on cardiovascular outcomes, long-term safety, and quality-of-life improvements to fully evaluate the benefits of efpeglenatide.

## Conclusions

Efpeglenatide advances the treatment of T2DM and obesity by improving blood sugar regulation, promoting weight loss, and supporting cardiovascular health. Its weekly dosing regimen enhances patient compliance and ease of use, addressing long-term illness management challenges. Studies have shown that efpeglenatide significantly reduces HbA1c levels (up to 1.5%) and body weight (average 4.5 kg over 26 weeks) in diverse patient groups, highlighting its effectiveness and broad applicability for personalized treatment. The side effects of efpeglenatide are similar to those of other GLP-1 RAs, with gastrointestinal issues (nausea, vomiting, and diarrhea) being the most common, typically mild to moderate and transient. Hypoglycemia is rare, particularly in patients not receiving insulin or sulfonylureas. Cardiovascular and renal benefits, such as fewer major adverse cardiovascular events and improved renal outcomes, are particularly observed in high-risk patients, making efpeglenatide a comprehensive treatment for T2DM and obesity that extends beyond glycemic control.

Limitations of the current research include a moderate risk of bias in some studies, primarily due to open-label designs and subjective outcome evaluations. The long-term effects on cardiovascular mortality, kidney function, and quality of life require further investigation. Real-world studies focusing on adherence, cost-effectiveness, and patient satisfaction are also required to validate efpeglenatide performance outside controlled trials. Overall, efpeglenatide demonstrates promising efficacy and safety, improved glycemic control, reduced body weight, and enhanced cardiovascular health in patients with T2DM and obesity. Weekly dosing improves treatment adherence and patient satisfaction. Future research should focus on long-term outcomes and comparative analyses with other treatments to confirm the role of efpeglenatide in the comprehensive management of diabetes and obesity.
